# Hydroxy-*α*-sanshool Possesses Protective Potentials on H_2_O_2_-Stimulated PC12 Cells by Suppression of Oxidative Stress-Induced Apoptosis through Regulation of PI3K/Akt Signal Pathway

**DOI:** 10.1155/2020/3481758

**Published:** 2020-07-09

**Authors:** Ruo-Lan Li, Qing Zhang, Jia Liu, Jia-yi Sun, Li-Ying He, Hu-Xinyue Duan, Wei Peng, Chun-Jie Wu

**Affiliations:** School of Pharmacy, Chengdu University of Traditional Chinese Medicine, Chengdu 611130, China

## Abstract

*Zanthoxylum bungeanum* pericarp is a commonly used herbal medicine in China with effects of anti-inflammatory and analgesic, improving learning and memory ability, while hydroxy-*α*-sanshool (HAS) is the most important active ingredient of *Z. bungeanum* pericarps. The purpose of this study was to investigate the neuroprotective effect of HAS and its related possible mechanisms using a H_2_O_2_-stimulated PC12 cell model. CCK-8 assay results showed that HAS had a significant protective effect on H_2_O_2_-stimulated PC12 cells without obvious cytotoxicity on normal PC12 cells. Flow cytometry and fluorescence microscope (DAPI staining and DCFH-DA staining) indicated that HAS could reduce the H_2_O_2_-induced apoptosis in PC12 cells via reduction of intracellular ROS and increase of mitochondrial membrane potential (MMP). Subsequently, results of malondialdehyde (MDA), superoxide dismutase (SOD), catalase (CAT), and glutathione peroxidase (GSH-Px) determination suggested that HAS could increase the enzyme activities of SOD, CAT, and GSH-Px whereas it could decrease the MDA contents in H_2_O_2_-stimulated PC12 cells. Furthermore, the western blotting assays showed that HAS could upregulate the expressions of p-PI3k, Akt, p-Akt, and Bcl-2, while it could downregulate the expressions of cleaved caspase-3 and Bax in H_2_O_2_-stimulated PC12 cells. Collectively, it could be concluded according to our results that HAS possesses protective potentials on H_2_O_2_-stimulated PC12 cells through suppression of oxidative stress-induced apoptosis via regulation of PI3K/Akt signal pathway.

## 1. Introduction

Increasing evidences have revealed that oxidative stress is closely related to neurodegenerative diseases, such as Parkinson's disease and Alzheimer's disease. In the body, excessive reactive oxygen species (ROS) is commonly considered the main cause corresponding to oxidative stress [1–3]. ROS, such as hydrogen peroxide (H_2_O_2_), superoxide anions, and hydroxyl radicals, can stimulate cells which cause structural damage including lipid peroxidation and DNA and protein oxidation, promote oxidative stress, and disrupt the redox balance of the body, as well as change the normal function and morphology of cells [4]. There are a variety of antioxidant systems in cells, while the synergistic antioxidant effect is mainly achieved by eliminating intracellular ROS to prevent oxidative damage to the body [5]. In fact, oxidant/antioxidant levels are critical for neurodegeneration or neuroprotection, in which enzymes such as superoxide dismutase (SOD), catalase (CAT), and glutathione peroxidase (GSH-Px) constitute the key antioxidant defenses [6]. Excessive ROS not only is closely related to mitochondrial dysfunction but also can increase intracellular Ca^2+^ concentration and activate some intracellular apoptotic pathways. Among them, the PI3K/Akt signaling pathway is closely correlated to it, which is also involved in the changes of Bcl-2 family proteins and the activation of caspase family proteins [7].

It is no doubt that herbal medicines are beneficial for treating various diseases with low toxic and side effects. *Zanthoxylum bungeanum*, belonging to the *Rutaceae* family, is a known medicinal plant widely distributed in China. *Z. bungeanum* pericarp is a known spice in China and widely used in cooking because of its unique fragrance and taste [8, 9]. According to the *Compendium of Materia Medica*, it can be used to treat various diseases such as vomiting, pathogenic wind, and toothache [10, 11]. In addition, modern pharmacological and phytochemical evidences have found that essential oil in *Z. bungeanum* pericarps has a variety of pharmacological effects, including antitumor effects, anti-inflammatory effects, and antibacterial and insecticidal activities [12–16]. In addition, the unsaturated fatty acid amides in *Z. bungeanum* pericarps, such as hydroxy-*α*-sanshool (HAS), hydroxy-*β*-sanshool, and hydroxy-*γ*-sanshool, also have wide-spectrum pharmacological activities, including hypolipidemic and hypoglycemic effects and anti-inflammatory and neurotrophic effects [17, 18]. Further studies have also shown that HAS has an antioxidant effect and can improve scopolamine-induced learning and memory impairments in rats [19, 20]. Consequently, we speculated that HAS may have neuroprotective potentials, and the present study was aimed at investigating the protective effect of HAS and its related possible mechanisms using a H_2_O_2_-stimulated PC12 cell model.

## 2. Materials and Methods

### 2.1. Materials and Chemicals

Hydroxy-*α*-sanshool (HAS) (purity was higher than 98%) used in the present study was isolated from the *Z. bungeanum* pericarps and supplied by the PUSH Bio-Technology (Chengdu, China). Fetal bovine serum (FBS) and horse serum (HS) were purchased from the Hyclone Co. (Logan, UT, USA). H_2_O_2_ was purchased from Chengdu Chron Chemicals Co. Ltd. (Chengdu, China). RPMI-1640 culture medium, phosphate-buffered saline (PBS), and 0.25% trypsin-EDTA (1x) were purchased from Gibco Co. (Grand Island, NY, USA). Dimethyl sulfoxide (DMSO), cell counting kit-8 (CCK-8), BCA protein assay reagents, and primary antibodies for Bcl-2, Bax, and cleaved (C) caspase-3 were purchased from Boster Biol. Tech. (Wuhan, China). Primary antibodies for PI3K, phosphorylation- (p-) PI3K, AKT, and p-AKT were obtained from the ImmunoWay Biotechnology Co. (Suzhou, China). The assay kits for DCFH-DA, MDA, and SOD and horseradish peroxidase- (HPR-) conjugated secondary antibody were purchased from the Beyotime Institute of Biotechnology (Haimen, China). The assay kits for LDH, CAT, and GSH-PX were purchased from the Nanjing Jiancheng Bioengineering Institute (Nanjing, China). The 5,5′,6,6′-tetrachloro-1,1′,3,3′-tetraethyl-imidacarbocyanine iodide (JC-1) was obtained from the Jiangsu KeyGen Biotech. (Nanjing, China). All other reagents used in the experiments were of analytical grade.

### 2.2. Cell Culture and Treatment

The PC12 cells were purchased from Wuhan Pu-nuo-sai Life Technology Co. Ltd. (Wuhan, China) and used throughout the study. PC12 cells were cultured in RPMI-1640 medium containing 5% FBS (*v*/*v*), 5% horse serum, penicillin (100 units/mL), and streptomycin (100 *μ*g/mL) at 37°C in a humidified atmosphere of 5% CO_2_. Cells were subcultured twice a week, and only those in the exponential growth phase were used in experiments.

PC12 cells were pretreated with different concentrations of HAS (15, 30, and 60 *μ*M) for 2 hours and then incubated with 90 *μ*M H_2_O_2_ for another 4 hours. The control group was administered with the same amount of 1640 medium and then stimulated with H_2_O_2_. HAS was solubilized with DMSO and subsequently diluted in culture medium with the final concentration of DMSO less than 0.1% (*v*/*v*).

### 2.3. Determination of Cell Viability

Cell counting kit-8 was used to test cell activity. Before the formal experiment, the cytotoxicity of HAS on PC12 cells was first investigated. Briefly, PC12 cells were cultured in 96-well plates with 1 × 10^4^ cells per well and incubated with PC12 cells with 6.5-120 *μ*M of HAS for 24 hours. Subsequently, CCK-8 solution was added to each well and cells were kept in a humidified atmosphere of 5% CO_2_ at 37°C for 1 hour. Finally, the optical density (OD) values at 450 mm were measured by a microplate reader (Bio-Rad, Hercules, CA, USA). After that, PC12 cells were pretreated with different concentrations of HAS (7.5-120 *μ*M) for 1-4 hours and then incubated with 90 *μ*M H_2_O_2_ for another 4 hours to select the optimal working concentration of HAS for the further experiments.

After selecting the optimal time and concentration of HAS, the cells were incubated at 37°C for 24 hours, pretreated with HAS (final concentrations in the well were 15, 30, and 60 *μ*M) for 2 hours, and then stimulated with H_2_O_2_ (final concentration was 90 *μ*M) for 4 hours. The control group was administered with the same amount of 1640 medium, while the positive group was incubated with 100 *μ*M vitamin C and then stimulated with H_2_O_2_.

### 2.4. Nuclear Staining with DAPI

PC12 cells were seeded in 6-well plates with a density of 1 × 10^5^ per well. The cells were incubated at 37°C for 24 hours and pretreated with HAS (final concentrations in the well were 15, 30, and 60 *μ*M) or 100 *μ*M vitamin C for 2 hours and then stimulated with H_2_O_2_ (final concentration was 90 *μ*M) for 4 hours. Subsequently, the cells were washed twice with PBS and then fixed with 10% paraformaldehyde in each well. After fixation, the cells were stained with DAPI solution, incubated at room temperature for 5 min, and then washed three times with PBS. Finally, the staining of the cells was observed under a fluorescence microscope (Olympus, IX-83, Tokyo, Japan).

### 2.5. Apoptosis Assay by Flow Cytometer

For the apoptosis of PC12 cells, the Annexin V/FITC kit was used. The cells were incubated in 6-well plates with a density of 1 × 10^5^ per well and given different concentrations of HAS (final concentrations in well were 15, 30, and 60 *μ*M) or 100 *μ*M vitamin C and H_2_O_2_ as described above. Subsequently, cells were collected and washed twice with PBS at 4°C, while the supernatant was removed by centrifugation. At the final concentration, the cells were suspended with 500 *μ*L binding buffer and incubated with 5 *μ*L Annexin V-FITC and 5 *μ*L PI for 15 minutes at room temperature; then a FACSCanto II Flow cytometer (BD Company, New York, NY, USA) was used to detect cell apoptosis.

### 2.6. Assessment of Mitochondrial Membrane Potential

The decrease of intracellular mitochondrial membrane potential (MMP, ΔΨ_m_) can be used as an important indicator of mitochondrial dysfunction, JC-1 is commonly considered as an ideal probe to evaluate ΔΨ_m_. At a hyperpolarized membrane potential, JC-1 aggregates in the mitochondrial matrix to form polymers that emit red fluorescence; while when it is at the depolarized membrane potential, JC-1 only emits green fluorescence as a monomer. Therefore, the fluorescence transformation will directly reflect the ΔΨ_m_ changes. Consequently, in our present study, PC12 cells were seeded in 6-well plates and treated as described in the individual experiment, then incubated with JC-1 at 37°C in the dark for 15 min. After washing the cells twice with PBS, the cells' fluorescence was measured by a using a laser confocal microscopy (Leica, SP8 SR, Wetzlar, Germany).

### 2.7. Detection of Intracellular ROS Accumulation in PC12 Cells

In an oxidized environment, DCFH-DA can be transformed into green fluorescent DCFH in the cell and the intracellular ROS could be monitored by fluorescent probe DCFH-DA. Briefly, cells were incubated in 6-well plates with different pretreatment or stimulation. Subsequently, the supernatant was removed and cells were incubated with DCFH-DA (10 *μ*M) for 20 min at 37°C in a dark environment and followed by washing for three times with PBS. Intracellular ROS was analyzed by measuring the fluorescence intensity of DCF with a FACSCanto II Flow cytometer (BD Company, New York, NY, USA).

### 2.8. Determination of MDA, SOD, GSH-Px, and CAT in H_2_O_2_-Induced PC12 Cells

The cells were incubated in 6-well plates and given different concentrations of HAS and H_2_O_2_ as described above. The supernatants were removed; then cells were washed with PBS for three times. Subsequently, the cells were lysed by lysis buffer, which was collected and centrifuged to obtain the total cell protein. Protein concentrations, MDA level, and activities of SOD, GSH-Px, and CAT were determined using commercial assay kits according to the manufacturer's instructions.

### 2.9. Western Blotting Assay

After treating as described in the individual experiment, cells were harvested and total proteins were extracted using RIPA lysis buffer. The protein concentrations were determined using BCA protein assay reagents; subsequently, the total protein (30 *μ*g) was separated by 12% SDS-PAGE, then transferred to the PVDF membrane. After blocking by sealing fluid (5% skimmed milk), the PVDF membrane was incubated overnight with diluted primary antibodies of C-caspase-3, Bax, Bcl-2, PI3K, p-PI3K, Akt, and p-Akt (dilution 1 : 1000), respectively, at 4°C. Subsequently, the PVDF membrane was incubated with HPR-conjugated secondary antibody (1 : 5000) at room temperature for 1 hour. Finally, the protein bands were stained with ECL detection kits, and *β*-actin was used as the internal reference. Image analysis software ImageJ (version 1.51, National Institutes of Health, MD, USA) was used for gray analysis.

### 2.10. Determination of Cell Viability after the Inhibition of Signaling Pathway

To further examine the role of the PI3K/Akt signaling pathway in HAS protecting PC12 cells from H_2_O_2_ stimulation, we used a chemical inhibitor LY294002 to inhibit the expression of the PI3K/Akt signaling pathway by CCK-8. In this part, the HAS group was incubated with 60 *μ*M HAS and 90 *μ*M H_2_O_2_, and the HAS+LY294002 group was pretreated with 20 *μ*M LY294002 for 1 hour and then incubated 60 *μ*M HAS and 90 *μ*M H_2_O_2_, while the LY294002 group was only treated with LY294002 and H_2_O_2_.

### 2.11. Statistical Analysis

Data are presented as the mean ± standard deviations (SD). Statistical comparisons except the seizure rate were made by Student's *t*-test or one-way analysis of variance (ANOVA) using GraphPad Prism 5 software (GraphPad Software Inc., La Jolla, CA). *P* < 0.05 was considered the significant level.

## 3. Results

### 3.1. HAS Protects the Cell Viability of H_2_O_2_-Stimulated PC12 Cells

**Figure 1:**
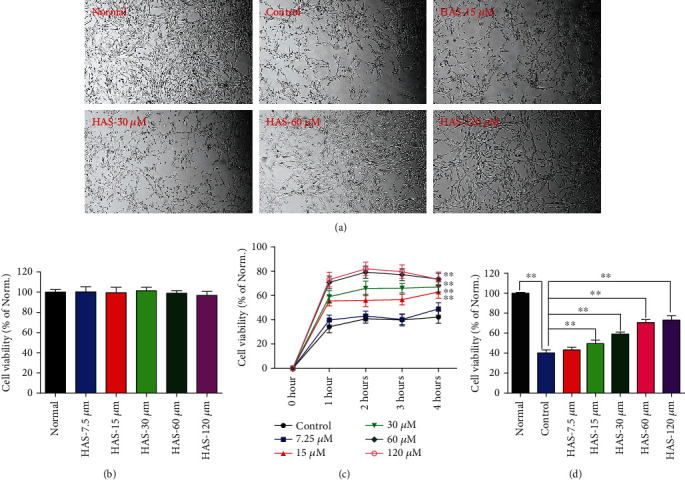
Protective effects of HAS on the cell viability of H_2_O_2_-stimulated PC12 cells. (a) The represented cell morphology of PC12 cells with different treatment (×100). (b) Effects of HAS on cell viability of normal PC12 cell. (c) Effects of HAS on cell viability of H_2_O_2_-induced PC12 cells under different concentration and time. (d) Effects of HAS pretreatment for 2 hours on cell viability of H_2_O_2_-induced PC12 cells. HAS: hydroxy-*α*-sanshool; Norm.: normal. The values were represented as the mean ± SD (*n* = 3). ^∗∗^*P* < 0.01 vs. the control group.

As can be seen from the Figure 1(b), HAS at the concentration ranging from 7.5 to 120 *μ*M had no obvious effects on the viability of PC12 cells and the viability of all groups was approximate. In addition, CCK-8 assay results showed that 90 *μ*M H_2_O_2_ treatment could significantly decrease the viability of PC12 cells, making them 40% lower than the normal group (*P* < 0.01) (Figures 1(a) and 1(d)). What is more, it can be seen from Figure 1(c) that the optimal working time for HAS was 2 hours. Importantly and interestingly, pretreatment with HAS (15, 30, 60, and 120 *μ*M) for 2 hours could significantly concentration-dependently increase the cell viability of H_2_O_2_-stimulated PC12 cells, compared to the control PC12 cells (*P* < 0.01) (Figures 1(a) and 1(d)).

### 3.2. HAS Suppresses Apoptosis in H_2_O_2_-Stimulated PC12 Cells

**Figure 2:**
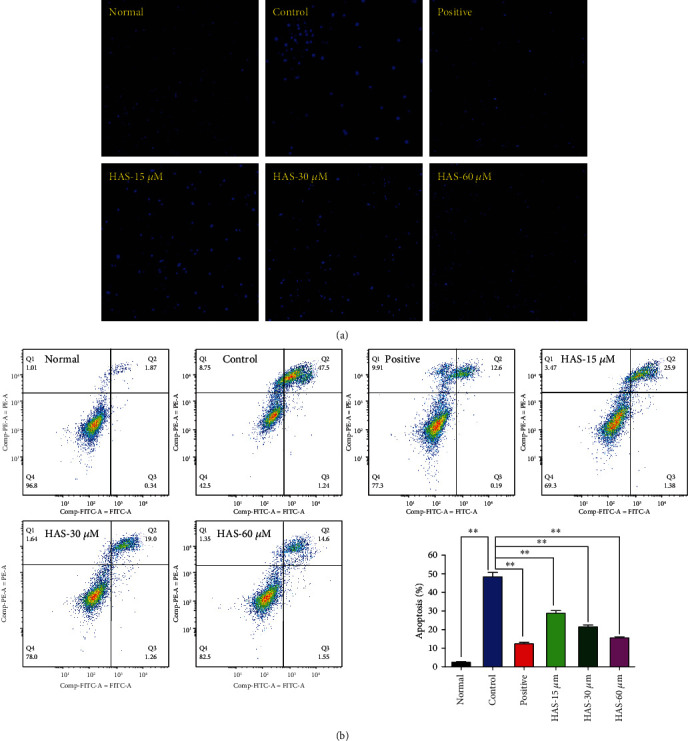
Effects of HAS on apoptosis in H_2_O_2_-stimulated PC12 cells. (a) Apoptotic assay by DAPI staining and observed under a 100x microscope. (b) Apoptotic assay by flow cytometry. Vitamin C (100 *μ*M) was used as the positive control. The values were represented as the mean ± SD (*n* = 3). ^∗∗^*P* < 0.01 vs. the control group.

To evaluate the apoptosis of PC12 cells, DAPI staining and flow cytometric assay with Annexin V-FITC/PI staining were utilized. As shown in Figure 2(a), nuclear morphological changes of the H_2_O_2_-stimulated PC12 cells were examined by staining with cell permeable DNA dye DAPI. For normal PC12 cells, PC12 cells were alive and the cell nucleus was round and intact with faint DAPI staining. However, after stimulation with 90 *μ*M H_2_O_2_ for 2 h, nuclear shrinkage or condensation and improved brightness could be obviously observed in the cell nucleus, indicating characteristic apoptotic features appeared. Interestingly, pretreatment with 100 *μ*M vitamin C or HAS (15, 30, and 60 *μ*M) significantly attenuated the apoptosis induced by H_2_O_2_ in PC12 cells, compared to the control PC12 cells. Besides that, we also found the protective effect of 60 *μ*M HAS was approximated to 100 *μ*M vitamin C.

Moreover, the further results of flow cytometric assay also confirmed the DAPI staining experiment. After induction with 90 *μ*M H_2_O_2_, the apoptosis rate of PC12 cells sharply increased to 48.74% compared with the normal group 2.21% (*P* < 0.01). However, pretreatment with 100 *μ*M vitamin C or different concentrations of HAS (15, 30, and 60 *μ*M) for 2 hours significantly improved the apoptosis induced by H_2_O_2_ stimulation (*P* < 0.01) with an obvious concentration-dependent manner, compared to the control PC12 cells (Figure 2(b)).

**Figure 3:**
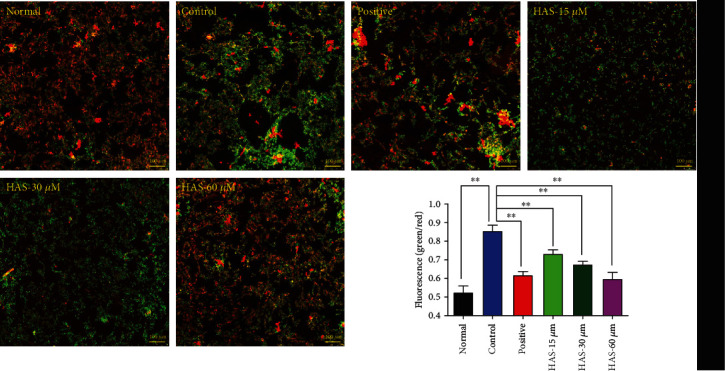
Effects of HAS on the ΔΨ_m_ in PC12 cells (×100). Cells were pretreated with vitamin C (100 *μ*M) or HAS (15, 30, and 60 *μ*M) for 2 h and then incubated in the presence of H_2_O_2_ (90 *μ*M) for 4 h. ΔΨ_m_ was measured using a JC-1 assay kit and observed using a laser confocal microscopy under a 100× microscope. HAS: hydroxy-*α*-sanshool. The values were represented as the mean ± SD (*n* = 3). ^∗∗^*P* < 0.01 vs. the control group.

Besides, we also used JC-1 probe to detect the loss of ΔΨ_m_ in PC12 cells exposed to H_2_O_2_. ΔΨ_m_ was determined according to the green/red fluorescence ratio in PC12 cells. As shown in Figure 3, after incubation with 90 *μ*M H_2_O_2_ for 2 h, the green fluorescence of the PC12 cells increased sharply, and the ratio of green/red fluorescence became more than 80%. All of these indicated an obvious decline of ΔΨ_m_. However, pretreatment with 100 *μ*M vitamin C or HAS (15, 30, and 60 *μ*M) reversed the green/red ratio significantly, while 60 *μ*M HAS could exploit the advantages to the full (Figure 3). All the above results suggested that HAS could significantly suppress H_2_O_2_-stimulated apoptosis in PC12 cells.

### 3.3. HAS Decreases the H_2_O_2_-Stimulated ROS Generation in PC12 Cells

**Figure 4:**
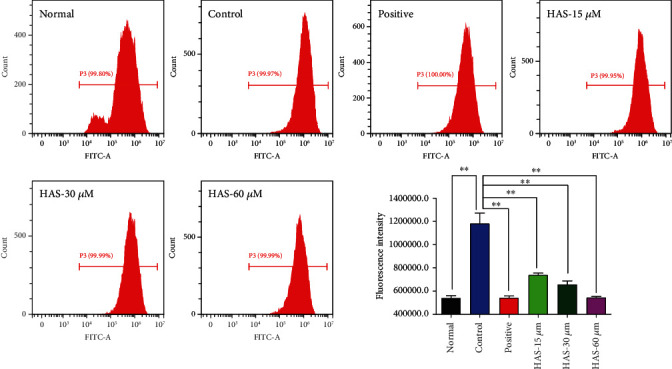
Effects of HAS on ROS levels of H_2_O_2_-stimulated PC12 cells. PC12 cells were treated with vitamin C (100 *μ*M) or HAS (5, 30, and 60 *μ*M) for 2 h, subsequently subjected to H_2_O_2_ (90 *μ*M) for 4 h. The intracellular ROS level was determined by the flow cytometry (FCM) assay. The values were represented as the mean ± SD (*n* = 3). ^∗∗^*P* < 0.01 vs. the control group.

Results of the above studies revealed that HAS could suppress H_2_O_2_ stimulation-induced apoptosis in PC12 cells. Importantly, cells stimulated by H_2_O_2_ will produce excessive ROS, which is also an important cause of cell apoptosis [5, 21]. To investigate the possible mechanisms for the antiapoptotic effects of HAS on H_2_O_2_-induced PC12 cells, we determined the ROS generation in PC12 cells. We used the fluorescence probe DCFH-DA to further explore whether HAS could prevent H_2_O_2_-stimulated ROS generation and resulting oxidative stress. As can be seen from the Figure 4, it was found that when the cells were exposed to 90*μ*M H_2_O_2_, the ROS produced in the cells increased sharply, compared to the normal cells (*P* < 0.01). However, pretreatment with 100*μ*M vitamin C or HAS (15, 30, and 60 *μ*M) significantly reduced the intracellular ROS accumulation in H_2_O_2_-induced PC12 cells, compared to the control cells (*P* < 0.01).

### 3.4. HAS Enhances the Activities of ROS Scavenging Enzymes in H_2_O_2_-Stimulated PC12 Cells

**Figure 5:**
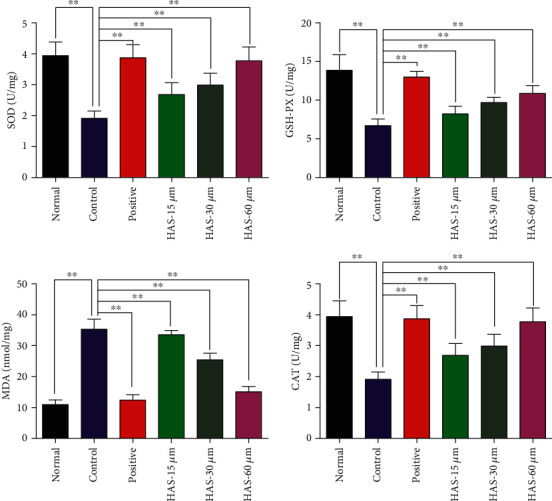
Effects of HAS on SOD, GSH-Px, MDA, and CAT in H_2_O_2_-stimulated PC12 cells. The levels of MDA and activities of SOD, CAT, and GSH-Px were determined by commercial assay kits. PC12 cells were treated with vitamin C (100 *μ*M) or HAS (15, 30, and 60 *μ*M) for 2 h, subsequently subjected to H_2_O_2_ (90 *μ*M) for 4 h. The values were represented as the mean ± SD (*n* = 3). ^∗∗^*P* < 0.01 vs. the control group.

Intracellular MDA, SOD, GSH-Px, and CAT are commonly used biomarkers for the evaluation of the oxidative stress level of cells or tissues [21–23]. Thus, to clarify whether HAS protects PC12 cells from H_2_O_2_ induced damage through antioxidant action or not, we studied the effect of HAS on MDA production and activities of ROS scavenging enzymes (SOD, GSH-Px, and CAT) in H_2_O_2_-stimulated PC12 cells. As shown in Figure 5, an extremely significant increase in MDA was observed in PC12 cells exposed to 90 *μ*M H_2_O_2_ for 2 hours (*P* < 0.01), compared to the normal cells. However, this growth trend was greatly alleviated by pretreatment with 100 *μ*M vitamin C or HAS (15, 30, and 60 *μ*M) for 2 hours (*P* < 0.01), compared to the control cells. On the other hand, the amount of antioxidant enzymes including SOD, GSH-Px, and CAT is reduced sharply in H_2_O_2_-stimulated PC12 cells (*P* < 0.01), compared with the normal cells. At the same time, the content of the three enzymes can be increased to different degrees by incubating the cells with HAS for 2 hours (*P* < 0.01). All the above results indicate that HAS treatment might be beneficial for protecting PC12 cells from the H_2_O_2_-caused damage through enhancement of activities of ROS scavenging enzymes.

### 3.5. HAS Regulates the Expressions of Caspase-3, Bax, and Bcl-2 in H_2_O_2_-Stimulated PC12 Cells

**Figure 6:**
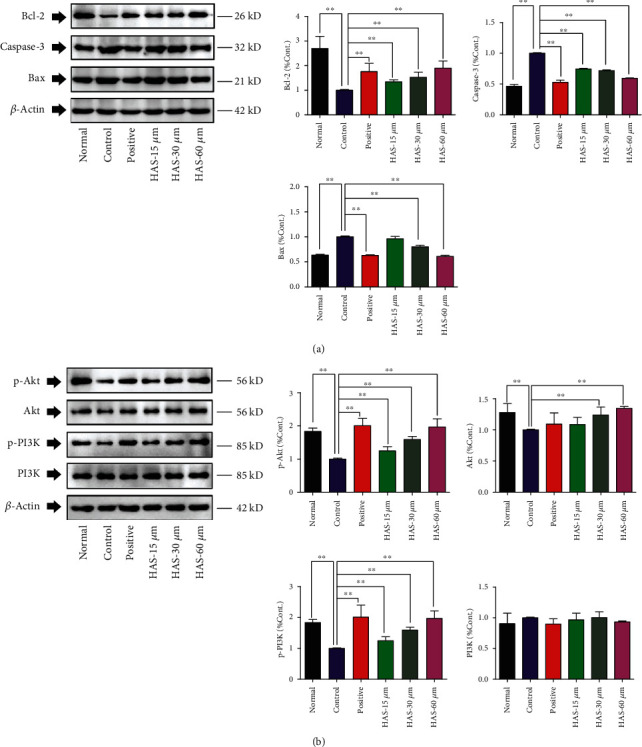
Effects of HAS on protein expressions of caspase-3, Bax, Bcl-2, PI3K, p-PI3K, Akt, and p-Akt in H_2_O_2_-stimulated PC12 cells. PC12 cells were treated with HAS (15, 30, and 60 *μ*M) or 100 *μ*M vitamin C for 2 h, subsequently subjected to H_2_O_2_ (90 *μ*M) for 4 h. Protein expressions of caspase-3, Bax, Bcl-2, PI3K, p-PI3K, Akt, and p-Akt were determined by western blotting, and *β*-actin was used as the internal reference. The values were represented as the mean ± SD (*n* = 3). ^∗∗^*P* < 0.01 vs. the control group.

In order to explore the molecular mechanism for antiapoptotic effects of HAS on H_2_O_2_-stimulated PC12 cells, western blotting assays were carried out to detect the expressions of caspase-3, Bax, and Bcl-2 in cells. The Bcl-2 family proteins are key regulatory factors in mitochondrial-mediated apoptosis, which are divided into two categories: proapoptotic proteins (Bax, Bim, Bak, etc.) and antiapoptotic proteins (Bcl-2, Bcl-xl, Mcl-1, etc.) [24]. As shown in Figure 6(a), compared to the normal cells, 90 *μ*M H_2_O_2_ stimulation significantly downregulated the antiapoptotic protein of Bcl-2 in PC12 cells (*P* < 0.01), while upregulating the proapoptotic proteins of Bax and cleaved casepase-3 (*P* < 0.01). However, the present results also showed that HAS (15, 30, and 60 *μ*M) and 100 *μ*M vitamin C could reverse abovementioned changes and upregulated Bcl-2 (*P* < 0.01) whereas they could downregulate caspase-3 in H_2_O_2_-stimulated PC12 cells (*P* < 0.01), compared to the control cells. Besides these, Bax could be downregulated by treatment with 100 *μ*M vitamin C or HAS at the concentrations of 30 and 60 *μ*M in H_2_O_2_-stimulated PC12 cells (*P* < 0.01), compared to the control cells.

### 3.6. HAS Regulates the Expressions of PI3K, p-PI3K, Akt, and p-Akt in H_2_O_2_-Stimulated PC12 Cells

The PI3K/Akt signal pathway is essential for the survival of neurons related to suppression of apoptosis [25]. In our present results as shown in Figure 6(b), it was found that expressions of Akt, p-Akt, and p-PI3K in PC12 cells were significantly decreased after stimulation with H_2_O_2_ for 2 hours (*P* < 0.01), compared to the normal cells. However, pretreatment with HAS (15, 30, and 60 *μ*M) and 100 *μ*M vitamin C significantly upregulated the p-Akt and p-PI3K in PC12 cells (*P* < 0.01), compared to the control cells. Besides, pretreatment with HAS (30 and 60 *μ*M) could also significantly upregulate Akt in H_2_O_2_-stimulated PC12 cells (*P* < 0.01), compared to the control PC 12 cells, while PI3K expression difference was not statistically significant. These results suggested that HAS may possess protective potentials on H_2_O_2_-stimulated PC12 cells via the PI3K/Akt pathway.

**Figure 7:**
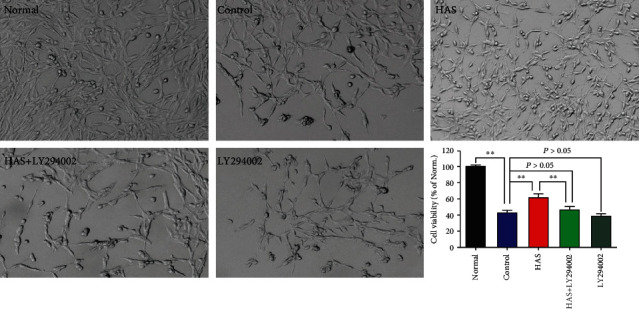
Effects of PI3K inhibitor LY294002 on the viability of PC12 cells (×100). The PC12 cells were pretreated with LY294002 (20 *μ*M) or not for 1 hour; then cells were treated with HAS (60 *μ*M) for 2 h, subsequently subjected to H_2_O_2_ (90 *μ*M) for 4 h. HAS: hydroxy-*α*-sanshool; Norm.: normal. The values were represented as the mean ± SD (*n* = 3). ^∗∗^*P* < 0.01 vs. the control group.

To further explore whether the PI3K/Akt pathway is the key in the protective effect of HAS, we used a chemical inhibitor LY294002 to inhibit the expression of the PI3K/Akt signaling pathway. As shown in Figure 7, after incubation with 90 *μ*M H_2_O_2_, the viability of PC12 cells dropped to nearly 40%; the LY294002 group was approximated to it. Besides that, the HAS group could increase the viability of PC12 cells to 60%, while the HAS/LY294002 group just increased a little. All of these data showed that HAS possessed protective potentials on H_2_O_2_-stimulated PC12 cells via the PI3K/Akt pathway.

## 4. Discussion

Hydroxy-*α*-sanshool (HAS) is a promising natural monomer of unsaturated fatty acid amide isolated from the *Z. bungeanum* pericarps with lots of bioactivities, such as hypolipidemic and hypoglycemic effects, improving learning and memory effects. As part of our continuing research on this compound, to the best of our knowledge, the present study provides the first evidence that HAS can protect PC12 cells from H_2_O_2-_induced damage through the suppression of apoptosis.

Previous researches revealed that PC12 cell, a rat photochromogenic cell line, has some neuronal characteristics and similar physiology and pathology of the nerve cells, and in addition, H_2_O_2_ possesses high cell membrane transmittance; consequently, H_2_O_2_-stimulated PC12 cells commonly considered an ideal cell model for studying pathology and screening candidate drugs of neurodegenerative diseases, such as Alzheimer's disease (AD) and epilepsy [26–28]. Thus, the PC12 cell line was selected as the experimental cell model in our present study. According to relevant research, many free radicals are generated during the development of neurodegenerative diseases, and some of the reactive oxygen species (ROS) can cause oxidative damage to nerve tissues and eventually lead to apoptosis or even necrosis of neurons [29, 30]. Therefore, ROS play an important role in the apoptosis caused by oxidative stress. As an important member of the ROS family, H_2_O_2_ can easily cross cell membranes to generate hydroxyl radicals, which are highly toxic and can cause serious damage to cells and attack biomolecules, ultimately leading to apoptosis or necrosis [31–33]. Therefore, in this study, H_2_O_2_ was used to stimulate PC12 cells to simulate the occurrence and development of neurodegenerative diseases caused by oxidative stress. According to CCK-8 assay, after stimulation with H_2_O_2_, cell viability of the PC12 cells decreased by 60%; however, pretreatment with HAS reversed the decrease of cell viability induced by H_2_O_2_.

For the neurodegenerative diseases, the excessive ROS can lead to direct oxidative damage of molecules, followed by cell dysfunction and apoptosis [5, 34]. Our present investigation found that H_2_O_2_ stimulation resulted in excessive ROS accumulation in PC12 cells, and interestingly, HAS pretreatment could decrease the excessive ROS in PC12 cells caused by H_2_O_2_. Malondialdehyde (MDA), a product of lipid peroxidation, would be significantly increased when exposed to oxidative stimulation, which is also considered a biomarker of oxidative stress and also causes damage to the cell membrane [22, 23]. In addition, there are various ROS scavenging enzymes in living organisms, among which the most important are the superoxide dismutase (SOD), catalase (CAT), and glutathione peroxidase (GSH-Px). Under physiological conditions, they jointly maintain the redox balance in the body [35]. In vivo, SOD can catalyze the conversion of superoxide anions into H_2_O_2_, GSH-Px can reduce toxic peroxides to nontoxic hydroxyl compounds, and CAT can promote the further conversion of H_2_O_2_ into oxygen and water [36, 37]. According to our results, H_2_O_2_-stimulated PC12 cells produced excessive MDA, accompanied by significantly decreased activity of SOD, GSH-Px, and CAT. Interestingly, pretreatment with HAS can decrease the MAD level whereas it can increase the activities of SOD, GSH-Px, and CAT in stimulated PC12 cells. In previous reports, excessive intracellular ROS produced by mitochondria could also lead to mitochondrial dysfunction through oxidative stress-induced apoptosis, and MMP is a sensitive indicator of mitochondrial function [38, 39]. In our results, after H_2_O_2_ stimulation, significant apoptosis and reduced cell survival as well as declined ΔΨ_m_ can be found in PC12 cells. In addition, intracellular ROS accumulation increased after H_2_O_2_ stimulation, which further promoted the loss of ΔΨ_m_. Besides, as expected, H_2_O_2_-induced cell apoptosis events in PC12 cells can be blocked by pretreatment with HAS. All these results suggested that HAS might be beneficial for protecting H_2_O_2_-stimulated PC12 cells from ROS-induced apoptosis.

**Figure 8:**
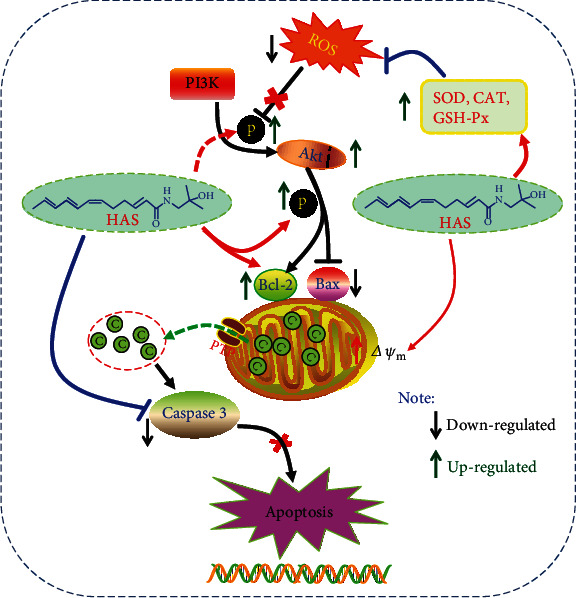
Molecular mechanism of the protective effect of HAS. HAS possesses protective potentials on H_2_O_2_-stimulated PC12 cells through suppression of oxidative stress-induced apoptosis via regulation of the PI3K/Akt signal pathway.

Currently, increasing evidences have suggested that the PI3K/AKT signal pathway plays a crucial role in cell survival and development as well as ROS-induced cell apoptosis [40, 41]. In addition, the PI3K/AKT pathway shows significant antioxidant activity in central and peripheral neurons and can be considered a potential therapeutic target for neurodegenerative diseases, participating in the cellular protective mechanism of ROS-induced cell damage [39]. AKT is a serine/threonine kinase activated by recruitment to the plasma membrane and is a key mediator of PI3K-mediated signal transduction [42, 43]. As is shown in Figure 8, the direct results of PI3K phosphorylation is the phosphorylation of AKT, which further affects the expression of Bcl-2 and Bax proteins. Bcl-2 and Bax are a group of proteins closely related to mitochondrial mediated apoptosis, among which the antiapoptotic protein Bcl-2 is a channel protein located on the mitochondrial membrane, which can inhibit the proapoptotic effect of Bax [44]. Activated p-AKT increased the expression of Bcl-2 and decreased the expression of Bax. In normal PC12 cells, these entire proteins in this signal pathway would be in a dynamic balance [45]. Our results showed that HAS pretreatment could upregulate the proteins of PI3K/Akt signaling (p-PI3K, Akt, and p-Akt) and antiapoptotic proteins of Bcl-2, whereas it could downregulate the apoptotic proteins (caspase-3 and Bax), compared to the PC12 without HAS treatment.

## 5. Conclusion

In summary, our study suggested that the hydroxy-*α*-sanshool (HAS) possesses protective potentials on H_2_O_2_-stimulated PC12 cells by suppression of oxidative stress-induced apoptosis through the regulation of the PI3K/Akt signal pathway. Our results provide scientific evidences that HAS might be considered in the development of a new drug for treating neurodegenerative diseases related to excessive apoptosis induced by oxidative stress.

## Data Availability

The data used to support the findings of this study are available from the corresponding author upon request.
